# Identification and functional characterization of the *RPP13* gene family in potato (*Solanum tuberosum* L.) for disease resistance

**DOI:** 10.3389/fpls.2024.1515060

**Published:** 2025-01-20

**Authors:** Baoqi Yuan, Chuang Li, Qingfeng Wang, Qi Yao, Xiaowei Guo, Yuhang Zhang, Zhongwei Wang

**Affiliations:** Institute of Economic Plants, Jilin Academy of Agricultural Sciences (Northeast Agricultural Research Center of China), Changchun, China

**Keywords:** potato, *RPP13* gene family, disease resistance, gene expression, subcellular localization

## Abstract

Potato (*Solanum tuberosum* L.), as the world’s fourth largest food crop, plays a crucial role in ensuring food security through its disease resistance. The *RPP13* gene family is known to play a pivotal role in plant disease resistance responses; however, its specific functions in potato remain unclear. In this study, we conducted the first comprehensive identification and analysis of 28 *RPP13* gene family members in potato, examining their gene structures, chromosomal locations, expression patterns, and functional characteristics. Gene structure analysis revealed that most members contain the typical CC-NBS-LRR domains, with exon numbers ranging from 1 to 6. Phylogenetic analysis grouped these genes into four evolutionary clades, indicating a high level of conservation. Cis-regulatory element analysis identified that the promoter region of *StRPP13-26* is enriched with pathogen-responsive elements such as the WUN-motif and MYC, suggesting its potential role in disease defense. Expression pattern analysis showed that *StRPP13-8*, *StRPP13-10*, and *StRPP13-23* are highly expressed in various tissues, indicating their involvement in basic physiological functions, whereas *StRPP13-6* and *StRPP13-25* are mainly induced under specific pathogen infection conditions. Transcriptome and qRT-PCR analyses further revealed functional divergence of the *RPP13* gene family in response to potato scab disease. Notably, *StRPP13-11* was significantly downregulated in both resistant and susceptible cultivars, suggesting its crucial role in the early stages of pathogen recognition. Subcellular localization experiments showed that the StRPP13-11 protein is localized in the chloroplast. Combined with transcriptome-based functional enrichment analysis, this finding implies that StRPP13-11 may participate in disease defense by regulating photosynthesis-related genes and the dynamic balance of reactive oxygen species within the chloroplast. This study provides new insights into the potential functions of the *RPP13* gene family in potato disease resistance mechanisms, offering valuable genetic resources and theoretical support for future disease-resistant breeding programs.

## 1 Introduction

Potato (*Solanum tuberosum* L.) is the fourth most important food crop globally, playing a critical role in ensuring food security due to its high yield, broad adaptability, and rich nutritional content (Ugent, 1970; Zaheer and Akhtar, 2016). As a staple food and significant economic resource for farmers, the potato’s high production potential and nutritional value make it a key crop for addressing food shortages, particularly in the face of rapid population growth and climate change (Devaux et al., 2014, Devaux et al., 2021; Dongyu, 2022).

However, the sustainable expansion of potato cultivation is increasingly threatened by various soil- and air-borne diseases, which significantly compromise yield and tuber quality (Visser et al., 2009; Zhang et al., 2017). To date, approximately 40 soil-borne diseases have been identified as major threats to potato production (Arora and Khurana, 2004). Among these, soil-borne diseases such as common scab (*Streptomyces scabies*), late blight (*Phytophthora infestans*), and black scurf (*Rhizoctonia solani*) are particularly devastating, causing global economic losses estimated in billions of dollars annually (Hill and Lazarovits, 2005; Tscharntke et al., 2012; Fiers et al., 2012; Arshaghi et al., 2023). Enhancing potato resistance to these diseases is therefore a pressing priority for ensuring agricultural sustainability and food security.

Resistance (R) genes are critical components of plant immunity, representing one of the largest and most diverse gene families in plants. These genes play pivotal roles in recognizing pathogen-derived effectors and triggering immune responses (Jin et al., 2024; Qi et al., 2024a). Among them, the *RPP13* gene family stands out for its ability to mediate pathogen-specific defense responses. In *Arabidopsis thaliana*, the *RPP13-Nd* gene was found to function independently of classical immune pathways like NDR1 and EDS1, highlighting its unique regulatory mechanism (Bittner-Eddy and Beynon, 2001). Furthermore, the high amino acid diversity of *RPP13* genes was reported to enhance their adaptability to evolving pathogens (Rose et al., 2004). In wheat, the *TaRPP13-3* gene, located on chromosome 7D and encoding a CC-NBS-LRR protein, was shown to confer resistance to powdery mildew, illustrating the potential of this gene family in crop protection (Liu et al., 2020). Zhang et al. (2022) further demonstrated that the truncated *TaRPP13L1-3D* gene positively regulated wheat resistance to powdery mildew via the RanGAP-WPP complex-mediated nucleocytoplasmic transport pathway. Recent studies also suggested that *RPP13*-like proteins were involved in abiotic stress responses, such as heat stress tolerance mediated by adenylyl cyclase activity in maize (Yang et al., 2021). These findings underscored the broad-spectrum functionality of *RPP13* genes, making them attractive targets for improving disease resistance in crops.

Despite these advances, the functional roles of the *RPP13* gene family in potatoes remain poorly understood. The lack of knowledge about their expression patterns, regulatory mechanisms, and specific contributions to disease resistance limits the ability to exploit these genes for breeding purposes. Addressing this gap is essential for leveraging *RPP13* genes in developing disease-resistant potato varieties (Ortega and Lopez-Vizcon, 2012; Paluchowska et al., 2022).

In this study, we conducted the first comprehensive identification and characterization of the *RPP13* gene family in potatoes. Specifically, we aimed to identify and annotate *RPP13* gene family members in the potato genome, investigate their gene structures, chromosomal distributions, and expression patterns, and validate the functional roles of key *RPP13* genes through subcellular localization experiments and transcriptomic analysis. These insights would pave the way for future molecular breeding programs, facilitating the development of potato varieties with enhanced disease resistance.

## 2 Results

### 2.1 Identification, chromosomal localization, and physicochemical properties of the *RPP13* gene family

**Figure 1:**
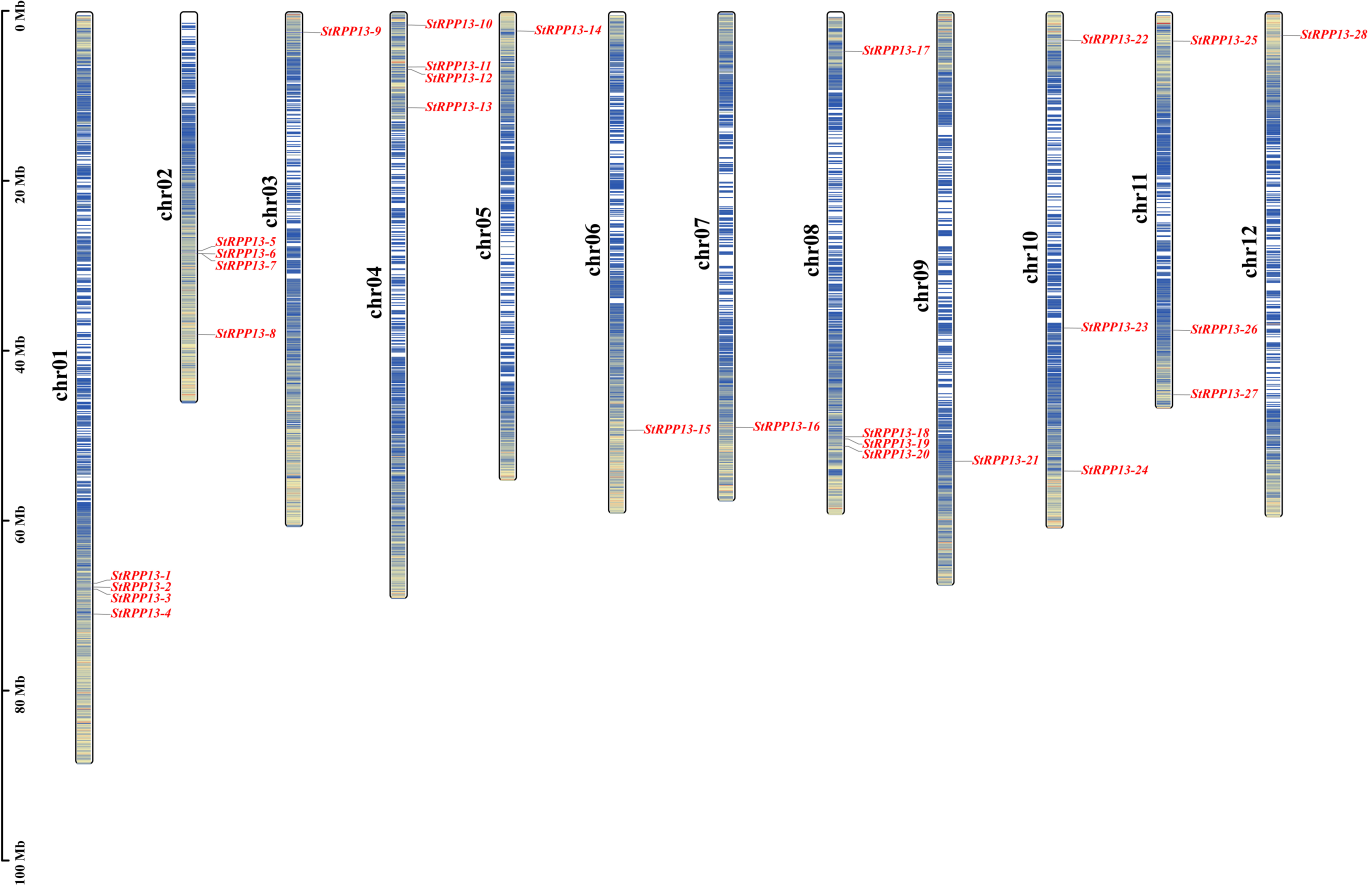
Chromosomal localization of the potato *RPP1*3 genes. The chromosome numbers were indicated on the left, with the scale on the left representing genetic distance in megabases (Mb). A genetic interval of 100 kb was used to precisely map the distribution of the *RPP13* genes. The color gradient from red to blue reflected gene density across the chromosomes, with darker regions indicating higher gene density and lighter regions representing lower gene density. Unmarked regions on the chromosomes denote areas for which no gene distribution data were available.

Using BLASTP analysis of the potato genome, 28 members of the *RPP13* gene family were identified and designated as *StRPP13-1* through *StRPP13-28*. These genes were distributed across 12 chromosomes (**Figure 1**), with chromosomes 1, 2, 4, and 8 each harboring four *RPP13* genes (e.g., *StRPP13-1* to *StRPP13-4* on chromosome 1). The remaining genes were distributed across other chromosomes, such as chromosomes 9 and 12, which also contained multiple *RPP13* genes (e.g., *StRPP13-18* to *StRPP13-20* and *StRPP13-25* to *StRPP13-28*). This distribution pattern suggested that the *RPP13* gene family exhibited a widespread and evolutionarily conserved organization within the potato genome.

The physicochemical properties of the RPP13 proteins revealed amino acid lengths ranging from 781 to 1714, with molecular weights varying between 89,918.09 Da and 195,237.26 Da. The theoretical isoelectric points (pI) ranged from 5.42 to 9.00, indicating a broad spectrum of acid-base properties. Most RPP13 proteins had instability indices below 50, suggesting that they were relatively stable within the cellular environment. Moreover, the GRAVY (Grand Average of Hydropathicity) values for all RPP13 family proteins were negative, reflecting their hydrophilic nature. Subcellular localization predictions indicated that all RPP13 proteins were localized in the cytoplasm (**Supplementary Table S1**).

### 2.2 Phylogenetic analysis of the *RPP13* genes

**Figure 2:**
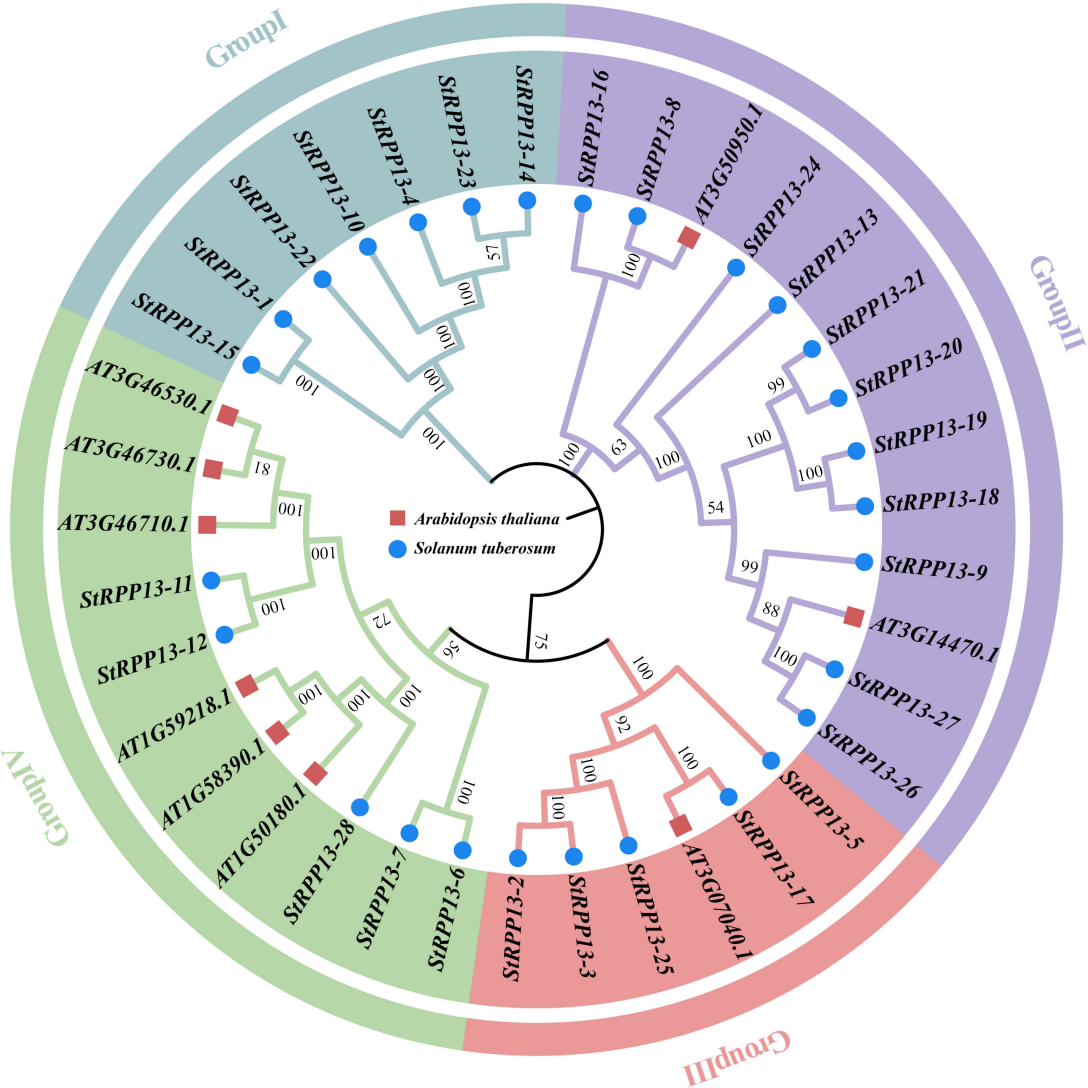
Phylogenetic analysis of potato and *Arabidopsis thaliana RPP13* gene family members. The phylogenetic tree divides the 28 potato *RPP13* genes and their *Arabidopsis* homologs into four groups. Potato *RPP13* genes were indicated by blue circles, while *Arabidopsis thaliana* homologs were marked with red squares.

A phylogenetic analysis of the *RPP13* gene family members from potato and *Arabidopsis thaliana* classified the 28 *RPP13* genes into four evolutionary clades (**Figure 2**). Group I included genes such as *StRPP13-1*, *StRPP13-4*, and *StRPP13-15*. Group II contained *StRPP13-18*, *StRPP13-9*, along with two *Arabidopsis* homologs (*AT3G50950.1* and *AT3G14470.1*), indicating strong evolutionary relationships. The members of Group III and Group IV demonstrated divergent evolutionary trajectories, with Group III genes such as *StRPP13-3* and *StRPP13-5* exhibiting unique conservation patterns. This classification revealed the evolutionary diversity of the *RPP13* gene family in potato, providing a foundation for future functional studies.

### 2.3 Gene structure, conserved motifs, and functional domain analysis

**Figure 3:**
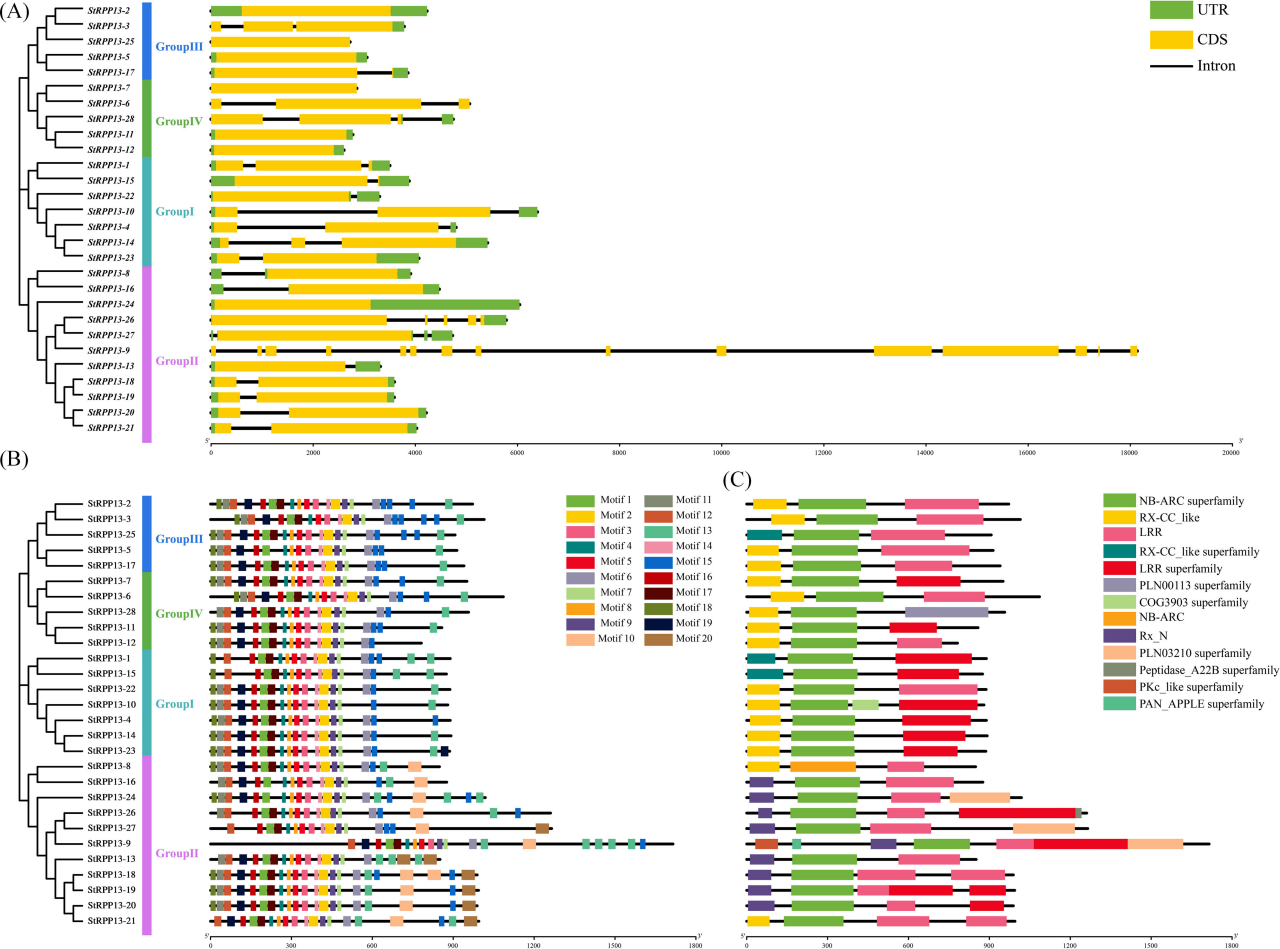
Gene structure, conserved motifs, and functional domains of potato *RPP13* genes. **(A)** The gene structures of *RPP13* family members, where green boxes represent untranslated regions (UTRs) at the 5’ and 3’ ends, yellow boxes represent coding sequences (CDS), and black lines represent introns. The numbers (0, 1, 2) indicate the intron phases. **(B)** Conserved motif patterns identified in the *RPP13* gene family. Different motifs are indicated by colored boxes, with each motif numbered and aligned according to their position within the gene. **(C)** Functional domain distribution of *RPP13* genes, where different colors correspond to distinct functional domains. The relative lengths of gene structures, motifs, and domains can be estimated using the scale bar at the bottom.

Gene structure analysis revealed that the number of exons in the *RPP13* genes ranged from 1 to 6. Most genes, such as *StRPP13-1* and *StRPP13-5*, contained 2 to 3 exons, while *StRPP13-26* exhibited a more complex gene structure with 6 exons, suggesting that this gene might possess intricate transcriptional and functional regulation mechanisms. Additionally, *StRPP13-9* was the longest gene in the family; despite having only 3 exons, it featured significantly extended introns, indicating that it might have unique regulatory characteristics (**Figure 3A**).

In the conserved motif analysis, 20 distinct conserved motifs were identified in the RPP13 proteins (**Figure 3B**). Motifs 1 through 4 were widely distributed across all RPP13 proteins, underscoring their evolutionary conservation. Motifs 16, 19, and 20 were predominantly present in Group III, suggesting their possible involvement in specific disease resistance functions. Functional domain analysis further confirmed the functional conservation of the RPP13 family proteins. All proteins were found to contain disease resistance-related domains such as NB-ARC, RX-CC, and LRR, with StRPP13-25 and StRPP13-26 in Group III also containing additional domains, including PLN00113 and the COG3903 superfamily domains, indicating that these proteins might play specialized roles in pathogen defense (**Figure 3C**).

### 2.4 Intraspecific and interspecific collinearity analysis of potato *RPP13* genes

**Figure 4:**
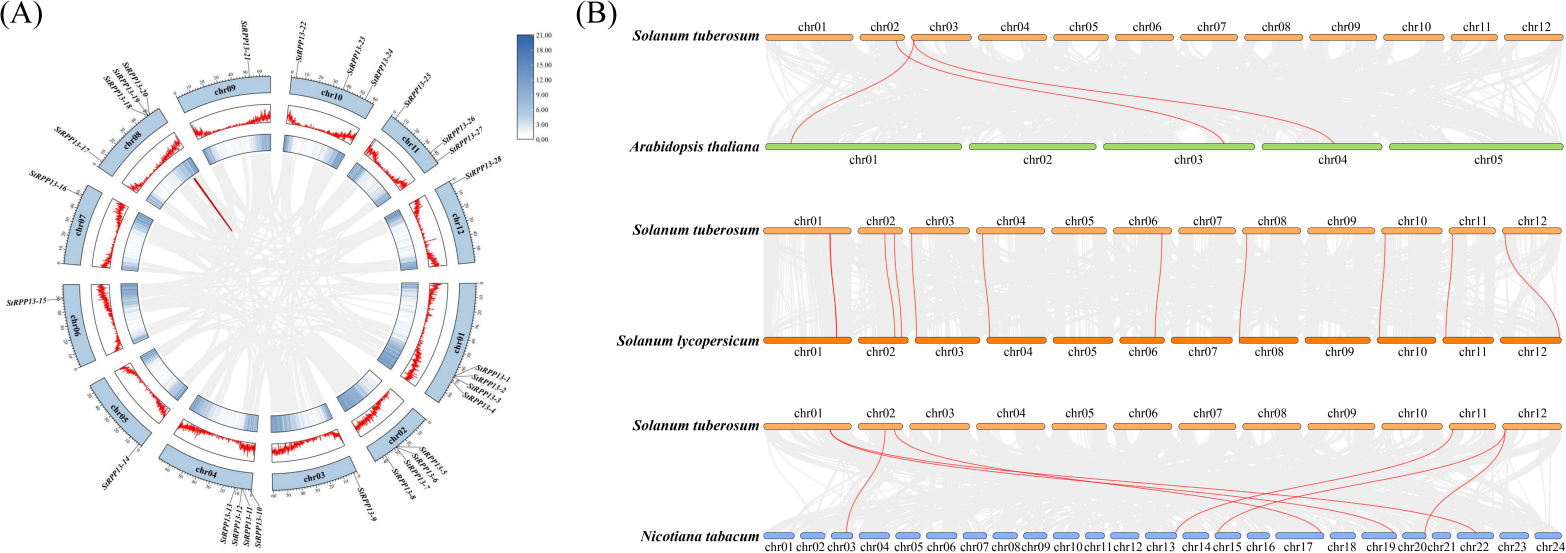
Collinearity analysis of the potato *RPP13* genes within and across species. **(A)** The intraspecific collinearity of the *RPP13* gene family within the potato genome. Collinear blocks across the entire genome were depicted with a grey background, while red curves connect pairs of duplicated *RPP13* genes, highlighting regions of segmental duplications. **(B)** Interspecific collinearity analysis between potato and other species (*Arabidopsis thaliana*, *Solanum lycopersicum*, and *Nicotiana tabacum*). Gray lines in the background represent collinear gene pairs between species, while red lines specifically connect collinear *RPP13* genes.

Collinearity analysis revealed the conservation and evolutionary relationships of the *RPP13* gene family both within and across species. Intraspecific collinearity analysis showed significant collinearity relationships of the *RPP13* genes on chromosomes 1, 2, 3, 8, 9, and 12. Particularly notable were the collinearity observed between chromosomes 2 and 3, and between chromosomes 8 and 9, suggesting the occurrence of tandem duplications or segmental duplications, which might have contributed to the expansion of this gene family. However, in the central chromosomal regions, collinearity between *StRPP13-19* and *StRPP13-20* was the only observed segment, indicating that these genes might have been subjected to specific selective pressures during evolution (**Figure 4A**).

Interspecific collinearity analysis revealed a degree of collinearity between the potato *RPP13* gene family and those in other Solanaceae species, such as tomato and tobacco, particularly on chromosomes 1, 2, and 12. This suggested that these gene families might have originated from a common ancestor and undergone conserved evolution within Solanaceae species. Specifically, synteny analysis with tomato suggested that these genes were highly aligned with chloroplast-related disease defense pathways, further supporting their conserved functional characteristics. In contrast, collinearity with *Arabidopsis thaliana* was limited, with only a few collinear segments observed on chromosomes 2 and 3. The collinearity with tobacco was more complex, indicating that multiple chromosomal rearrangement events might have occurred during evolution (**Figure 4B**).

### 2.5 Cis-acting element analysis of promoters in potato *RPP13* genes

**Figure 5:**
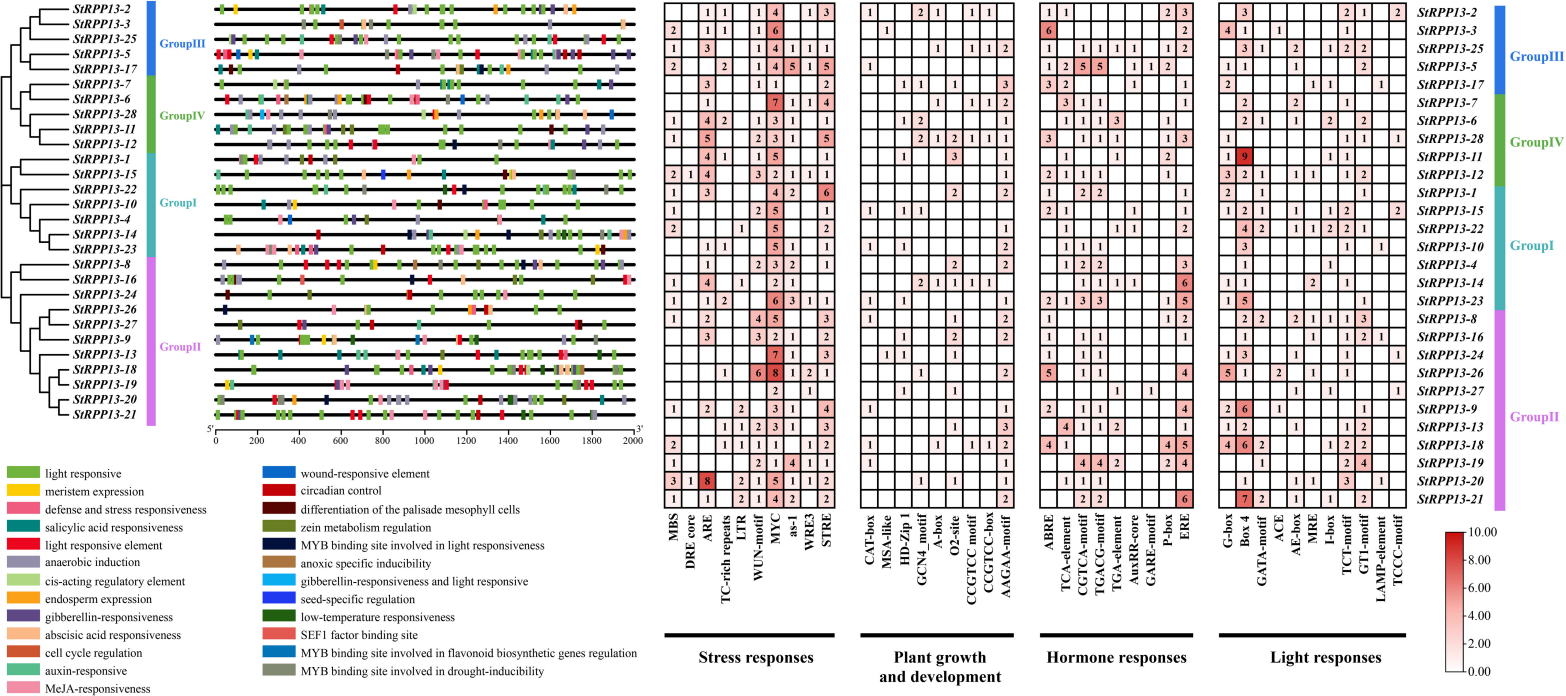
Cis-acting element analysis of *RPP13* gene promoters in potato. The diagram on the left illustrated the distribution of various cis-acting elements across the 2000 bp upstream regions of the 28 *RPP13* genes. Each colored box represented different functional elements, including light-responsive (green), meristem expression (light brown), defense and stress responsiveness (red), and hormone-responsive elements (orange), among others. On the right, heatmaps showed the abundance of stress response, plant growth and development, hormone response, and light response elements. Numbers within the boxes indicated the number of occurrences of each element in the respective *RPP13* gene promoter. The color gradient from light pink to dark red represents the frequency of each element, with darker colors indicating higher abundance.

Cis-acting element analysis of the promoter regions (2000 bp upstream) of 28 potato *RPP13* genes revealed the presence of multiple elements associated with environmental stress responses, plant growth and development, hormone responses, and light responses (**Figure 5**). Among these, stress-responsive elements such as MYC, STRE, and ARE were particularly prominent, with the highest abundance observed in *StRPP13-5*, *StRPP13-20*, and *StRPP13-26*. Additionally, *StRPP13-26* contained the largest number of WUN-motif, suggesting its involvement in wound response and pathogen defense mechanisms.

Plant growth and development-related elements, such as AAGAA-motif, O2-site, and GCN4-motif, were widely distributed among the genes, particularly in *StRPP13-22* and *StRPP13-15* in Group I and Group IV, indicating that these genes might play crucial roles in cell division and tissue differentiation.

Hormone response elements, such as ERE and ABRE, were highly enriched in *StRPP13-14*, *StRPP13-21*, and *StRPP13-3*, suggesting their involvement in hormone-regulated processes. Light response elements, including Box 4, G-box, and GT1-motif, were significantly increased in *StRPP13-18*, *StRPP13-20*, and *StRPP13-21*, indicating that these genes might function in light-regulated growth processes.

The cis-acting elements identified in the promoters of the *RPP13* gene family highlighted their critical roles in potato’s disease resistance, environmental stress response, hormone regulation, and light-responsive processes. Furthermore, the analysis suggested that these genes not only participated in disease defense mechanisms but might also influence the plant’s light response by regulating photosynthesis-related pathways and chloroplast metabolism, providing a more comprehensive view of their function in both defense and broader physiological processes.

### 2.6 Expression pattern analysis and qRT-PCR validation of *RPP13* genes in potato

#### 2.6.1 Tissue-specific expression analysis

Using the gene expression tools from the EnsemblPlants database, the expression patterns of 28 *RPP13* genes were analyzed across 14 different tissues, revealing significant variation in expression levels across tissues (**Supplementary Figure 1**). *StRPP13-8*, *StRPP13-10*, and *StRPP13-23* exhibited high expression levels in all tissues, suggesting their involvement in basic physiological processes across various potato tissues. In contrast, *StRPP13-6*, *StRPP13-7*, and *StRPP13-25* were nearly undetectable in most tissues, while *StRPP13-18*, *StRPP13-19*, *StRPP13-26*, and *StRPP13-27* displayed low expression across all tissues, indicating their potential roles under specific stress conditions.

Further analysis revealed that *StRPP13-2* and *StRPP13-24* showed significant expression in the root, tuber sprout, and whole *in vitro* plant, implying that these genes might be crucial in root development and tuber formation. *StRPP13-4* was exclusively expressed in the young tuber, while *StRPP13-15* and *StRPP13-28* were highly expressed only in the petiole. These findings suggested that the *RPP13* gene family displayed clear tissue specificity during various developmental stages and in different tissues of the potato, highlighting their potential roles in regulating diverse biological functions.

#### 2.6.2 Expression changes in resistant and susceptible cultivars post-pathogen infection

**Figure 6:**
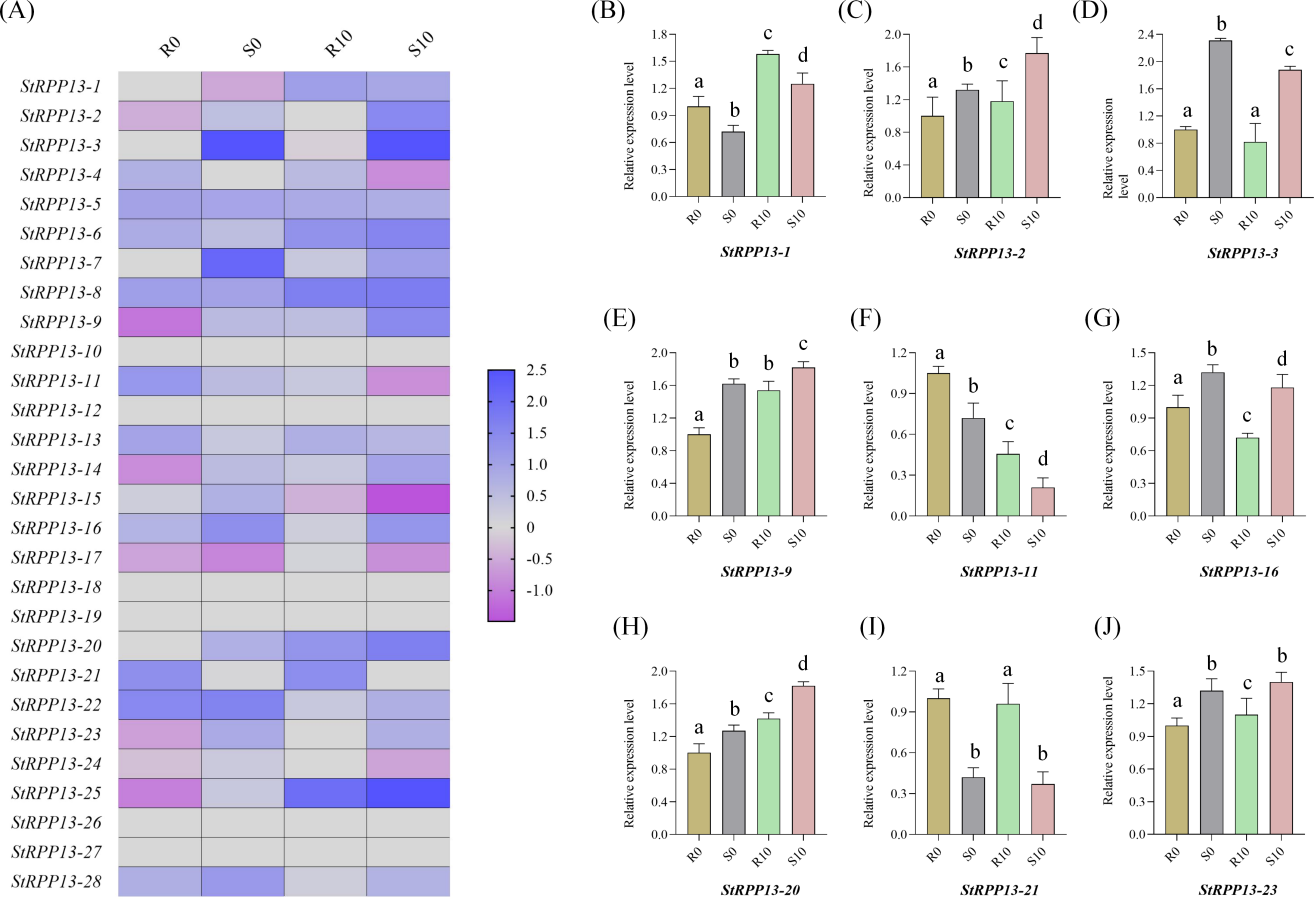
Expression pattern and qRT-PCR validation of the potato *RPP13* gene family in resistant (Chunshu 10) and susceptible (Chunshu 11) cultivars after *Streptomyces scabies* infection. **(A)** Heatmap showing the expression levels of 28 *RPP13* genes at 0 days (R0, S0) and 10 days (R10, S10) post-inoculation in resistant (R) and susceptible (S) cultivars. Expression values were represented as log2-transformed relative fold changes, with blue indicating higher expression and pink indicating lower expression. **(B–J)** qRT-PCR validation of selected *RPP13* genes in resistant (R0, R10) and susceptible (S0, S10) cultivars at 0 and 10 days post-inoculation. Data were presented as the mean ± standard error of three biological replicates. The expression levels were normalized against a housekeeping gene, and the relative fold change in expression was shown for each condition. Different letters indicated statistically significant differences among groups (*p* < 0.05, Duncan’s multiple range test).

Based on previous transcriptomic data, the expression changes of *RPP13* genes in resistant cultivar Chunshu 10 and susceptible cultivar Chunshu 11 were analyzed after inoculation with the pathogen *Streptomyces scabiei* at 0 and 10 days post-inoculation (**Figure 6A**). Results showed significant differences in the expression of certain *RPP13* genes between the resistant and susceptible cultivars. For example, the expression of *StRPP13-22* decreased significantly in the resistant cultivar 10 days post-inoculation, while no noticeable change was observed in the susceptible cultivar.

On the other hand, *StRPP13-16* exhibited a marked downregulation in the susceptible cultivar post-infection, suggesting its involvement in the pathogen response of the susceptible plant. Notably, *StRPP13-11* showed significant downregulation in both resistant and susceptible cultivars following pathogen infection, indicating that this gene might play a critical role in the early response to pathogen attack and could be a key regulator of resistance mechanisms. These results suggested that different members of the *RPP13* gene family exhibited diverse response patterns during the potato’s defense against scab disease.

#### 2.6.3 qRT-PCR validation

To further validate the expression patterns observed in the transcriptomic data, nine genes were randomly selected from the 28 *RPP13* genes for qRT-PCR analysis. The results showed that the expression changes of these genes in resistant and susceptible cultivars after *Streptomyces scabiei* infection were highly consistent with the transcriptomic data (**Figures 6B–J**). For instance, *StRPP13-2* and *StRPP13-3* exhibited significant expression differences between resistant and susceptible cultivars, particularly *StRPP13-11* and *StRPP13-21*, which showed marked downregulation 10 days post-inoculation. These findings further confirmed the differential expression patterns observed in the transcriptomic data, emphasizing their potential roles in the defense against disease.

### 2.7 Subcellular localization of the StRPP13-11 protein

To further investigate the specific subcellular localization of the key disease resistance gene *StRPP13-11*, a transient expression system using *Arabidopsis* protoplasts was employed. The *Arabidopsis* protoplast system, known for its efficient transient expression, allowed for the rapid evaluation of gene subcellular localization. The subcellular localization of the StRPP13-11 protein was determined by observing the GFP signal from the fusion protein under a laser confocal microscope.

**Figure 7:**
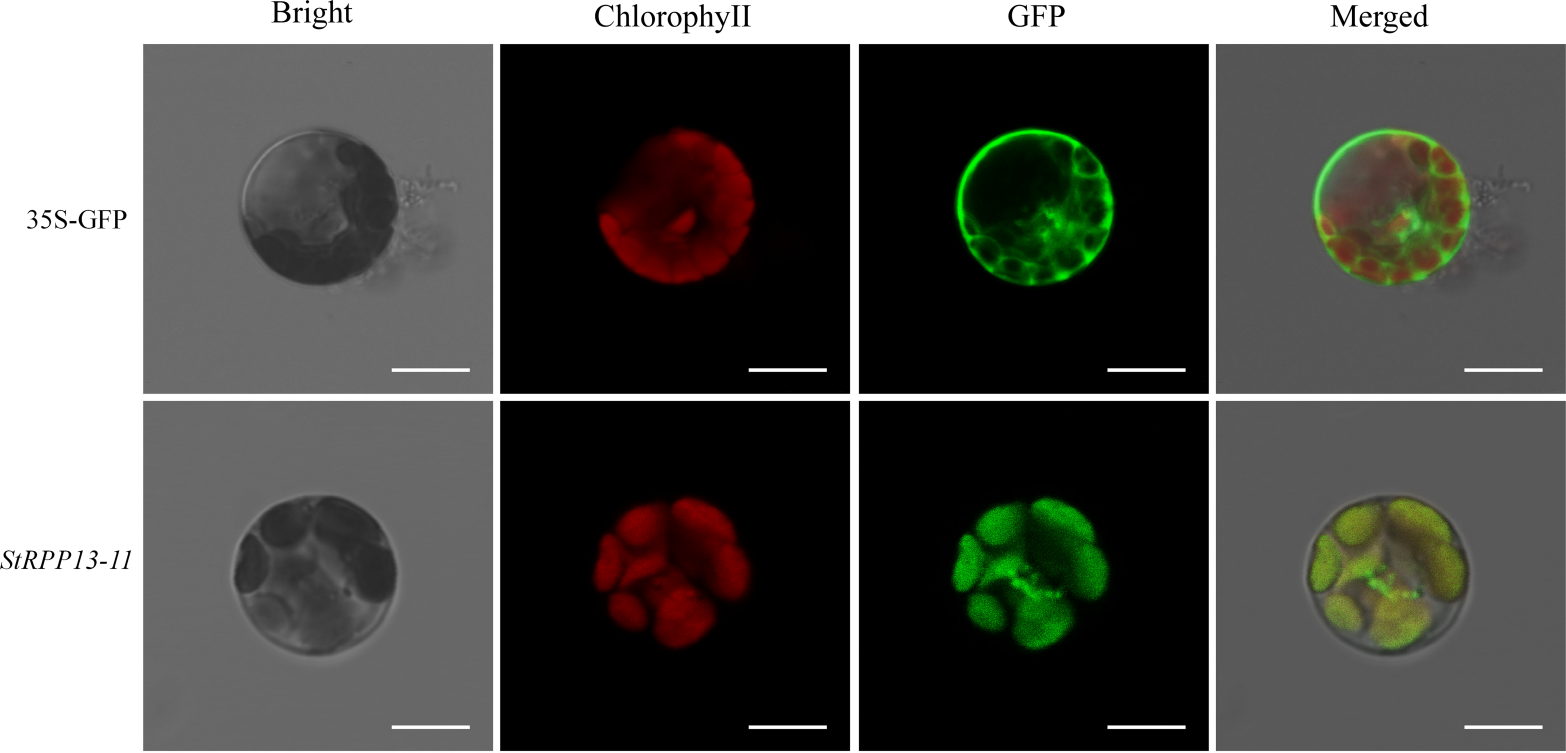
Subcellular localization of StRPP13-11 protein in *Arabidopsis* protoplasts using GFP fusion proteins. The control GFP (35S-GFP) was uniformly distributed throughout the cytoplasm. StRPP13-11-GFP fusion proteins showed a distinct GFP signal localized to the chloroplasts, co-localizing with the red autofluorescence of chlorophyll. The images in the “Bright” column represent bright field microscopy, the “Chlorophyll” column showed chlorophyll autofluorescence (red), the “GFP” column shows the GFP signal (green), and the “Merged” column presents an overlay of the GFP and chlorophyll signals. Scale bars = 10 µm.

The results showed that the GFP control (35S-GFP) was evenly distributed in the cytoplasm, whereas the GFP signal of the StRPP13-11-GFP fusion protein was mainly concentrated in the chloroplast region (**Figure 7**), with a high overlap with the red autofluorescence of chlorophyll. This localization result indicated that the StRPP13-11 protein is localized in the chloroplast. Chloroplasts are essential organelles in plant cells, responsible for photosynthesis and serving as hubs for numerous metabolic and signaling pathways. The chloroplast localization of StRPP13-11 suggested that it might exert direct influence on pathogen infection by regulating photosynthesis or chloroplast function as part of its defense mechanisms. This finding provided an important clue for further investigating its specific functions within the chloroplast.

## 3 Discussion

The *RPP13* gene family, as a typical class of CC-NBS-LRR resistance genes, plays a crucial role in plant disease resistance (van der Biezen and Jones, 1998). Its functional diversity and conservation are essential for understanding the mechanisms of plant immunity. In this study, we systematically identified 28 members of the *RPP13* gene family in potatoes and revealed their evolutionary characteristics through phylogenetic analysis and chromosomal localization. The results indicated that segmental and tandem duplication events were the main driving forces behind the expansion of this gene family, consistent with findings in other species (Cannon et al., 2004; Cheng et al., 2018). Compared to diploid species such as *Arabidopsis* and tomato, the *RPP13* gene family in potatoes exhibited greater diversity, which might be related to its complex tetraploid genome structure and specific environmental selection pressures (Meyers et al., 2003).

Phylogenetic analysis showed that most potato *RPP13* genes were conserved within the Solanaceae family, particularly displaying collinearity with homologous genes in tomato and tobacco (Huang et al., 2005). This suggested that the *RPP13* gene family might have originated from a common ancestor in these species and retained basic functional characteristics throughout evolution (Marla, 2017). However, some genes, such as *StRPP13-14* and *StRPP13-22*, lacked clear homologs in other species, indicating that these genes might have undergone species-specific expansion in potatoes and acquired novel functional differentiation in disease resistance (Bakker et al., 2006). Further analysis of gene structure and conserved motifs revealed that most members of the *RPP13* family possessed the typical CC-NBS-LRR domains, which is consistent with their roles in immune responses (Song et al., 2003). Despite experiencing gene expansion and diversification, the *RPP13* gene family has maintained its core functions (Kuang et al., 2005). Nevertheless, certain gene domains have undergone variations, possibly associated with specific functional differentiation and environmental adaptation (van der Vossen et al., 2005). This combination of conservation and diversity provides a multi-layered molecular basis for potatoes to cope with various pathogenic stresses.

This study also provides a preliminary analysis of the functional divergence and disease resistance mechanisms of the *RPP13* gene family in potatoes across different tissues and under the infection of the scab pathogen *Streptomyces scabies*. Cis-acting element analysis revealed that the promoter regions of genes such as *StRPP13-5*, *StRPP13-20*, and *StRPP13-26* were enriched with pathogen-responsive elements, including MYC, STRE, and ARE, with *StRPP13-26* showing the highest abundance of the WUN-motif (Rushton et al., 2010). This suggested that *StRPP13-26* might play a crucial role in wound response and pathogen defense, a finding consistent with the pathogen recognition mechanisms of *RPP13* genes in *Arabidopsis* and other species but highlighting a potentially unique pathogen-responsive function in potatoes (Meyers et al., 2003).

Gene expression pattern analysis indicated that *StRPP13-8*, *StRPP13-10*, and *StRPP13-23* were highly expressed across all tissues, suggesting their involvement in fundamental physiological processes. In contrast, *StRPP13-6*, *StRPP13-7*, and *StRPP13-25* showed minimal expression in most tissues, implying that these genes might be induced specifically under certain pathogen infection conditions (Sekhwal et al., 2015; Tang et al., 2017). This result differed from the expression characteristics of *RPP13* genes in diploid species such as *Arabidopsis* and rice. In *Arabidopsis*, *RPP13* genes were uniformly expressed across various tissues even under non-stress conditions, indicating their widespread role in immune surveillance (Mohr et al., 2010). In rice, the homologous *RPP13* genes were predominantly expressed in leaves and roots, particularly showing strong responses under pathogen infection (Nandety et al., 2013). In contrast, some *RPP13* genes in potatoes were activated only under specific pathogen infection conditions, reflecting a unique spatiotemporal regulatory pattern that might be linked to the tetraploid genome structure and the complex pathogen adaptation mechanisms of potatoes.

Transcriptome and qRT-PCR analyses further revealed the functional divergence of the *RPP13* gene family in potato resistance to scab disease. For instance, *StRPP13-22* showed significant downregulation in resistant cultivars after inoculation, while no substantial changes were observed in susceptible cultivars, suggesting its involvement in negative regulation of disease resistance. Meanwhile, *StRPP13-11* exhibited pronounced downregulation in both resistant and susceptible cultivars, indicating its potential role in the early stages of pathogen recognition. The presence of a high density of W-box cis-acting elements in its promoter suggested that *StRPP13-11* might interact with WRKY transcription factors to regulate downstream defense genes (Deslandes et al., 2003; Tsuda and Katagiri, 2010; Kuang et al., 2004). These findings suggested that the functional specificity of the potato *RPP13* gene family might stem from its unique evolutionary background and regulatory mechanisms.

Additionally, *StRPP13-11* was found to be localized in the chloroplast through subcellular localization experiments, suggesting that the chloroplast might play a crucial role in the disease resistance function of this gene family. Chloroplasts are not only the primary sites for photosynthesis and the production and scavenging of reactive oxygen species (ROS), but they also play a vital role in plant defense against pathogen infections (Song et al., 2021). Transcriptome analysis further supported this hypothesis, as photosynthesis-related pathways (photosynthesis pathway and chloroplast thylakoid membrane pathway) were significantly enriched in both GO and KEGG enrichment analyses, which aligned with the chloroplast localization of *StRPP13-11*. This suggested that the RPP13 proteins localized in the chloroplast might regulate plant defense responses by modulating the expression of photosynthesis-related genes and maintaining the dynamic balance of ROS in the chloroplast, thereby exerting their disease resistance function (De Torres Zabala et al., 2015; Li et al., 2024).

Recent research by Qi et al. (2024b) showed that the chloroplast elongation factors StTuA/B enhanced potato resistance to late blight and significantly increased crop yield by promoting chloroplast protein synthesis and photosynthesis efficiency. This finding highlighted the central role of chloroplasts in balancing growth and immunity and emphasized the importance of chloroplast pathways in potato disease defense, which was consistent with the chloroplast localization of *StRPP13-11* observed in our study. It suggested that the RPP13 proteins in the chloroplast might interact with WRKY transcription factors to regulate the expression of downstream defense genes, thus playing a role in pathogen recognition and resistance response.

The comprehensive identification and functional characterization of the *RPP13* gene family in potatoes revealed their critical roles in disease resistance, particularly against scab disease caused by *Streptomyces scabies*. Among the 28 identified genes, *StRPP13-11* emerged as a key candidate, localized in the chloroplast and potentially involved in regulating photosynthesis and ROS homeostasis. This study not only established a robust theoretical foundation for breeding potato varieties with enhanced disease resistance but also highlighted the potential of *RPP13* genes in breeding programs aimed at developing disease-resistant potato varieties. Future research should focus on validating the roles of key genes, such as *StRPP13-11*, and exploring their practical applications in crop improvement strategies.

## 4 Conclusion

This study presented the first comprehensive identification and characterization of the *RPP13* gene family in potatoes, revealing 28 members with conserved CC-NBS-LRR domains and grouping them into four evolutionary clades through phylogenetic analysis. Functional characterization highlighted their roles in potato disease resistance, particularly against scab disease. Notably, *StRPP13-11* was identified as a key gene, localized in the chloroplast and potentially involved in regulating photosynthesis and ROS homeostasis, underscoring its importance in early pathogen recognition. These findings offered a robust theoretical foundation and valuable genetic resources for future molecular breeding programs to enhance potato resistance to scab disease and other pathogens.

## 5 Materials and methods

### 5.1 Identification, physicochemical properties prediction, and chromosomal localization of potato *RPP13* genes

The potato genome data and its gff annotation file (DM 1-3 516 R44) were downloaded from the Spud DB online database (http://spuddb.uga.edu/). The amino acid sequences of *Arabidopsis thaliana RPP13* genes were obtained from the TAIR database (https://www.arabidopsis.org/) and used as references for BLASTP analysis. The specific locus names of the *Arabidopsis RPP13* genes used in this study are *AT1G59218.1*, *AT1G58390.1*, *AT3G46530.1*, *AT3G46710.1*, *AT3G46730.1*, *AT1G50180.*1, *AT3G07040.1*, *AT3G50950.1*, and *AT3G14470.1*. A local BLASTP analysis was conducted using the Diamond software (E-value set to 10^^-3^). The presence of characteristic domains of the RPP13 proteins, such as RX-CC, NB-ARC, and LRR, was confirmed using the Conserved Domain Database (CDD) of the National Center for Biotechnology Information (NCBI).

The physicochemical properties of the potato RPP13 proteins, including amino acid sequence length, theoretical isoelectric point (pI), molecular weight (Mw), instability index, aliphatic index, and grand average of hydropathicity (GRAVY) index, were calculated using the ProtParam tool (https://web.expasy.org/protparam/). Additionally, the subcellular localization of the RPP13 proteins was predicted using the CELLO online tool (http://cello.life.nctu.edu.tw/). Finally, the chromosomal locations of the *RPP13* genes were extracted from the genome annotation file and visualized using the Gene Location Visualize from GTF/GFF module of the Tbtools software (v2.097).

### 5.2 Phylogenetic, gene structure, and conserved motif analysis of potato *RPP13* genes

The amino acid sequences of RPP13 proteins from potato and *Arabidopsis thaliana* were aligned using the Clustal algorithm. A phylogenetic tree was constructed using the Neighbor-Joining (NJ) method in MEGA11 software with 1000 bootstrap replicates to assess branch confidence. The constructed phylogenetic tree was visualized and enhanced using the iTOL tool (https://itol.embl.de/). Bootstrap values are displayed at the corresponding nodes to indicate the confidence level of each branch.

The conserved motifs of the *RPP13* genes were analyzed using the MEME suite (http://meme-suite.org/) with the maximum number of motifs set to 20, the minimum width to 6, and the maximum width to 50. Gene structure analysis was performed by extracting the coding sequences (CDS) and untranslated regions (UTR) of the potato *RPP13* genes, and the results were visualized using TBtools v2.101.

### 5.3 Collinearity analysis of potato *RPP13* genes

The BLASTP program was employed to identify homologous *RPP13* genes in potato (E-value threshold < e^-5^). The collinearity relationships among potato *RPP13* genes were analyzed using the MCScanX program, and the collinear blocks and duplicated gene pairs were visualized using TBtools.

Additionally, genome sequences and annotation files of *Arabidopsis thaliana*, tobacco, and tomato were downloaded from the Phytozome v13 (https://phytozome-next.jgi.doe.gov/), NCBI (https://www.ncbi.nlm.nih.gov/), and Sol Genomics Network (https://solgenomics.net/organism/solanum_lycopersicum/genome) websites, respectively. Collinearity between potato and *Arabidopsis thaliana*, tobacco, and tomato was analyzed using MCScanX, and the results were visualized with TBtools.

### 5.4 Cis-regulatory element analysis in the promoters of potato *RPP13* genes

The 2000 bp sequences upstream of the start codon of each potato *RPP13* gene were extracted and considered as the promoter regions. The promoter sequences were extracted using TBtools and analyzed using the PlantCARE database (https://bioinformatics.psb.ugent.be/webtools/plantcare/html/) to predict cis-regulatory elements. The cis-regulatory elements associated with stress response, plant growth and development, hormone response, and light responsiveness were summarized and visualized using the Basic Biosequence View and HeatMap modules of TBtools.

### 5.5 Expression pattern analysis and qRT-PCR validation of potato *RPP13* genes

The expression patterns of 28 *RPP13* genes in 14 different tissues, including stolon, young tuber, flower, leaf, shoot apex, petiole, stem, mature tuber, root, tuber peel, tuber cortex, tuber pith, tuber sprout, and whole *in vitro* plant, were analyzed using the gene expression tool from the EnsemblPlants database (https://plants.ensembl.org/Solanum_tuberosum/Info/Index), and the results were visualized to show the expression differences across tissues.

Based on previous transcriptome sequencing data from our team, the expression changes of *RPP13* genes were analyzed in the resistant cultivar Chunshu 10 and the susceptible cultivar Chunshu 11 after inoculation with scab pathogen *Streptomyces scabies* at 0 days and 10 days post-inoculation (De Torres Zabala et al., 2015). The two cultivars were grown in pots, and a spore suspension of 2×10^^7^ spores/mL was applied during the sprouting phase (Wanner, 2007). Samples were collected at 0 days (pre-inoculation) and 10 days post-inoculation, rapidly frozen in liquid nitrogen, and stored at -80°C for further analysis.

To further validate the expression patterns of *RPP13* genes under scab infection, nine genes from the 28 *RPP13* family members were randomly selected for qRT-PCR analysis. Primer design was performed using Primer 3 (https://primer3.ut.ee/), and all primer sequences used in this study are provided in **Supplementary Table S2**. Total RNA was extracted from the samples using the TaKaRa MiniBEST Plant RNA Extraction Kit, and cDNA was synthesized using PrimeScript™ RT Master Mix (TaKaRa) (Ye et al., 2018). qRT-PCR was performed on the Applied Biosystems QuantStudio 6 system, with *StActin97* used as the reference gene. Each gene’s expression level was determined using three biological replicates, and relative expression levels were calculated using the 2^−ΔΔCT^ method (Livak and Schmittgen, 2001).

### 5.6 Subcellular localization

The subcellular localization of the *StRPP13-11* gene was analyzed using the *Arabidopsis* protoplast transient expression system. The *StRPP13-11* gene was cloned into the 16318-hGFP vector, and seamless cloning was used to transfer the gene into *Escherichia coli* DH5α for cloning and plasmid extraction. The extracted plasmid was then introduced into *Arabidopsis* protoplasts via PEG-mediated transformation, followed by washing and purification with W5 solution. The transformed protoplasts were incubated overnight (Kobayashi et al., 2006).

After transformation, the subcellular localization of the StRPP13-11 protein was observed using a laser confocal microscope at excitation wavelengths of 405 nm and 488 nm to detect GFP fluorescence (Zhu et al., 2023).

### 5.7 Statistical and data analysis

Statistical analyses were performed using Microsoft Excel 2013 and SPSS 19.0. Gene expression data from qRT-PCR experiments and transcriptomic analyses were included in the analysis. Group comparisons were carried out using Duncan’s multiple range tests to evaluate differences among experimental groups. To ensure the validity of ANOVA results, assumptions of normality and homogeneity of variances were verified using the Shapiro-Wilk test and Levene’s test, respectively. Only data that met these assumptions were subjected. For repeated experiments, the mean values were calculated from three biological replicates, and data variability was expressed as the standard deviation (SD). Significant differences among groups were indicated with different letters. A p-value of less than 0.05 was considered statistically significant. Data visualization, including graphical representations of gene expression levels, was conducted using GraphPad Prism 9.

## Data Availability

The datasets presented in this study can be found in online repositories. The names of the repository/repositories and accession number(s) can be found here: https://www.ncbi.nlm.nih.gov/, accession number PRJNA1057135.
