# Supplementary material for: Identification and functional characterization of the RPP13 gene family in potato (Solanum tuberosum L.) for disease resistance
Source: Front Plant Sci. 2025 Jan 20;15:1515060. doi: 10.3389/fpls.2024.1515060 (PMC11788377; doi:10.3389/fpls.2024.1515060)
Supplement: Supplementary file 1 [file DataSheet1.docx]

Supplementary Material

# Supplementary Figures


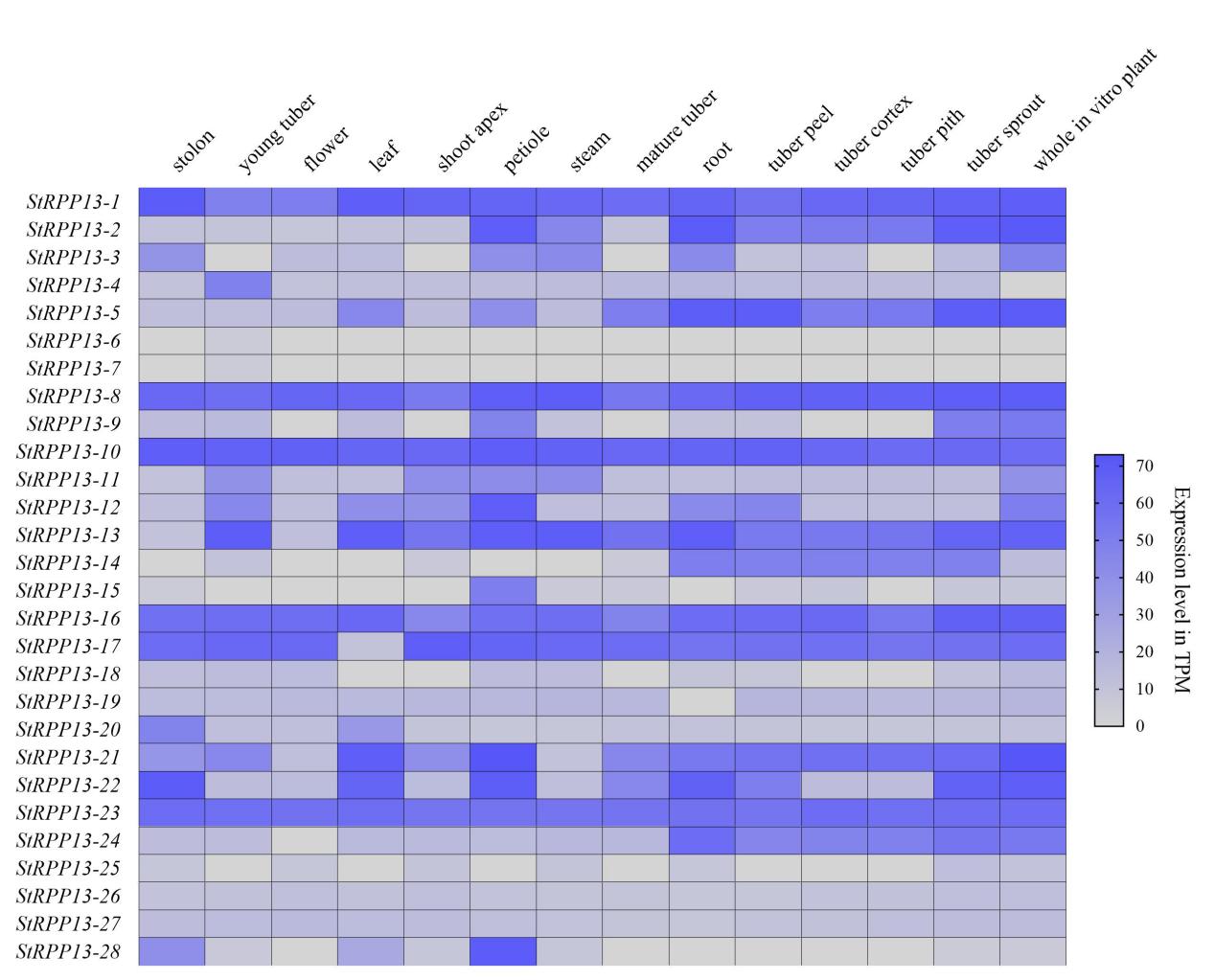


**Supplementary Figure 1.** Expression profiles of *RPP13* gene family in 14 different potato tissues. The heatmap displays the transcript levels (TPM) of 28 *RPP13* genes across various tissues, including stolon, young tuber, flower, leaf, shoot apex, petiole, stem, mature tuber, root, tuber peel, tuber cortex, tuber pith, tuber sprout, and whole in vitro plant. The intensity of blue shading corresponds to expression levels, with darker shades representing higher expression.

# Supplementary Tables

**Supplementary Table S1**.Characteristics of gene structures and protein properties of potato *RPP13* family members.

| Gene ID | Number of Amino Acids | Molecular Weight | Theoretical pI | Instability Index | Aliphatic Index | Grand Average of Hydropathicity | Subcellular localization prediction |
| --- | --- | --- | --- | --- | --- | --- | --- |
| *StRPP13-1* | 888 | 100750.6467 | 5.693527794 | 48.38502252 | 101.3175676 | -0.149436937 | Cytoplasmic |
| *StRPP13-2* | 971 | 111537.2269 | 6.846847725 | 43.65820803 | 105.3450051 | -0.135427394 | Cytoplasmic |
| *StRPP13-3* | 1014 | 116493.3357 | 7.549661827 | 44.94271203 | 104.4477318 | -0.137080868 | Cytoplasmic |
| *StRPP13-4* | 888 | 102106.0742 | 8.730453682 | 41.48231982 | 97.31981982 | -0.32027027 | Cytoplasmic |
| *StRPP13-5* | 913 | 104856.9714 | 8.745088005 | 47.56748083 | 102.2782037 | -0.155750274 | Cytoplasmic |
| *StRPP13-6* | 1085 | 123336.5376 | 7.931045723 | 47.09927189 | 106.7281106 | -0.131520737 | Cytoplasmic |
| *StRPP13-7* | 950 | 109321.9846 | 9.003670692 | 45.88982105 | 104.8315789 | -0.176210526 | Cytoplasmic |
| *StRPP13-8* | 848 | 96633.57798 | 6.624720955 | 44.61722877 | 95.17688679 | -0.234787736 | Cytoplasmic |
| *StRPP13-9* | 1714 | 195237.2626 | 5.687502861 | 44.60823804 | 95.59509918 | -0.144982497 | Cytoplasmic |
| *StRPP13-10* | 879 | 99889.00848 | 8.276016045 | 39.07088737 | 99.67007964 | -0.192377702 | Cytoplasmic |
| *StRPP13-11* | 857 | 98449.81408 | 6.801888084 | 43.90946324 | 103.3605601 | -0.152625438 | Cytoplasmic |
| *StRPP13-12* | 781 | 89918.08508 | 7.527096748 | 45.90590269 | 103.8028169 | -0.147631242 | Cytoplasmic |
| *StRPP13-13* | 850 | 96993.42488 | 7.012362862 | 44.63695294 | 103.6352941 | -0.119058824 | Cytoplasmic |
| *StRPP13-14* | 891 | 101478.2278 | 7.746608925 | 38.54971942 | 93.77104377 | -0.337934905 | Cytoplasmic |
| *StRPP13-15* | 874 | 100258.3184 | 8.351508522 | 44.41098398 | 100.3432494 | -0.223684211 | Cytoplasmic |
| *StRPP13-16* | 875 | 100676.6639 | 6.268852043 | 46.16262857 | 101.4742857 | -0.282057143 | Cytoplasmic |
| *StRPP13-17* | 939 | 107420.8417 | 8.742380333 | 41.35017039 | 98.4771033 | -0.220021299 | Cytoplasmic |
| *StRPP13-18* | 988 | 112380.294 | 7.140250587 | 45.38107287 | 99.55465587 | -0.232793522 | Cytoplasmic |
| *StRPP13-19* | 993 | 112760.6391 | 6.979225731 | 46.26686808 | 101.0271903 | -0.202215509 | Cytoplasmic |
| *StRPP13-20* | 988 | 112357.8882 | 7.29195385 | 45.57339069 | 100.2530364 | -0.193218623 | Cytoplasmic |
| *StRPP13-21* | 994 | 112738.9254 | 8.202973366 | 51.05202213 | 100.7947686 | -0.177162978 | Cytoplasmic |
| *StRPP13-22* | 887 | 101754.8275 | 8.431320381 | 43.82695603 | 97.33934611 | -0.220293123 | Cytoplasmic |
| *StRPP13-23* | 886 | 101325.4822 | 8.752566338 | 41.97608352 | 97.81038375 | -0.284537246 | Cytoplasmic |
| *StRPP13-24* | 1018 | 116863.5924 | 5.61560154 | 42.89656189 | 103.654224 | -0.166208251 | Cytoplasmic |
| *StRPP13-25* | 906 | 103970.568 | 7.157870674 | 40.56758278 | 103.4878587 | -0.119315673 | Cytoplasmic |
| *StRPP13-26* | 1260 | 143050.9791 | 5.501639366 | 38.3900873 | 99.11111111 | -0.18 | Cytoplasmic |
| *StRPP13-27* | 1264 | 143682.193 | 5.420700645 | 49.60087816 | 104.6281646 | -0.185443038 | Cytoplasmic |
| *StRPP13-28* | 956 | 111158.7175 | 8.786412239 | 43.65849372 | 93.28451883 | -0.345083682 | Cytoplasmic |

**Supplementary Table S2**.The primers used for qRT-PCR of nine potato *RPP13* family members.

| Primer | Forward Primer Sequence (5' to 3') | Reverse primer Sequence (5' to 3') |
| --- | --- | --- |
| *StRPP13-1* | CACCTTGTCAAACTGGTGCG | TCGATCAGTCAGAATTTCGAGC |
| *StRPP13-2* | ACTTACTCTGGCTGTCTTCTGC | TTCTTGGATACGGAGTCGCC |
| *StRPP13-3* | GGGACAGTTGAACACGTTGC | CCATGCATTGACAGCCATCG |
| *StRPP13-9* | TGCAAAGGCTGTCGATTCCT | CAAAGTGTCTGGCAGTTGGC |
| *StRPP13-11* | TCCAAGAAGCCGCTCCATATC | GACGACGGATACCACCTGTC |
| *StRPP13-16* | GGTCTGCAAAACTTCTCGTCA | CCCGTTTTTGGGCGACTTTT |
| *StRPP13-20* | TGAAGCCAAGTCCTCGACAC | GTTTGTGTGGGTTTGCCCTG |
| *StRPP13-21* | AAGCCTCTACGCGTGAAACA | AGCTGTACTAGATGCACCAACA |
| *StRPP13-23* | AGTACTCCCTTCGTCCATCT | TTCCCTCCAAGCCTCCAAAC |
